# Cryptogamia in the Roots of Hairs

**Published:** 1842-11-05

**Authors:** 


*Cryptogamia in the Roots of Hairs.*—M. Gruby presented to the Academy of Medicine, Paris, a memoir on a new species of cryptogame, which occupies the roots of the beard, and forms a species of contagious mentagra. The disease generally occupies the chin, lips, or cheeks ; the affected parts are covered with greyish and yellow scales, formed by the epidermic cells, under which is the root of the hair surrounded completely by a sheath of cryptogamia ; the latter are not elevated above the surface of the epidermis.—*Ibid.*

